# Presence and Cybersickness in Virtual Reality Are Negatively Related: A Review

**DOI:** 10.3389/fpsyg.2019.00158

**Published:** 2019-02-04

**Authors:** Séamas Weech, Sophie Kenny, Michael Barnett-Cowan

**Affiliations:** ^1^Department of Kinesiology, University of Waterloo, Waterloo, ON, Canada; ^2^The Games Institute, University of Waterloo, Waterloo, ON, Canada

**Keywords:** presence, cybersickness, virtual reality, sensory integration, human factors

## Abstract

In order to take advantage of the potential offered by the medium of virtual reality (VR), it will be essential to develop an understanding of how to maximize the desirable experience of “presence” in a virtual space (“being there”), and how to minimize the undesirable feeling of “cybersickness” (a constellation of discomfort symptoms experienced in VR). Although there have been frequent reports of a possible link between the observer’s sense of presence and the experience of bodily discomfort in VR, the amount of literature that discusses the nature of the relationship is limited. Recent research has underlined the possibility that these variables have shared causes, and that both factors may be manipulated with a single approach. This review paper summarizes the concepts of presence and cybersickness and highlights the strengths and gaps in our understanding about their relationship. We review studies that have measured the association between presence and cybersickness, and conclude that the balance of evidence favors a negative relationship between the two factors which is driven principally by sensory integration processes. We also discuss how system immersiveness might play a role in modulating both presence and cybersickness. However, we identify a serious absence of high-powered studies that aim to reveal the nature of this relationship. Based on this evidence we propose recommendations for future studies investigating presence, cybersickness, and other related factors.

## Introduction

Around 30 years ago, the process of simulating a user’s sensory environment gained the popular term “virtual reality”^1^ (Krueger, 1992). Although the concept of virtual reality (VR) has morphed significantly since the initial conception, the promise inherent in simulating “the real world” has continually inspired and challenged scientists and artists (Jones, 2000). Fifty years ago, when the first attempts to implement a VR display were taking place, a large number of technical issues required a solution in order to achieve even a rudimentary mediated environment. While working at Harvard Computation Laboratory, Ivan Sutherland’s team was able to solve many of these issues (Sutherland, 1968). Their stereoscopic display, including a refresh rate of 30 frames per second, a field-of-view of 40°, and the ability to depict 3D objects only as wire-frames, was termed “favorable” by users. This implementation was some distance from providing the idealized VR experience. Since these initial inventions, a vast amount of effort has been focused on the development of improved means of inspecting and interacting with virtual worlds, and a myriad of other problems have since been solved. This rapid progress has led to the creation of VR systems that are orders of magnitude smaller, lighter, and more powerful than Sutherland’s foundational technology. VR has recently seen popularity as a flexible tool for investigating a wide-range of human behaviors in high-fidelity with perfectly replicable conditions. Throughout the development process, however, the ultimate goal for VR has remained unachievable – that is, the accurate and credible simulation of a real experience. Chief among the enduring problems that prevent this achievement is the struggle to consistently generate a sense of *presence* in VR users, whereby conscious awareness of simulated mediation ceases. A second prominent barrier is cybersickness^2^ (CS), the bodily discomfort associated with exposure to VR content. Unlocking the potential of VR will largely depend on our ability to understand, then to solve, these substantial and enduring hindrances. A large body of research has emerged from attempts to identify whether presence and CS are deterministically linked through a positive or negative association. However, these results are highly discordant, and no consensus currently exists regarding the nature of the relationship between CS and presence.

This review has three aims. In the first part of this article we describe in brief the concepts of presence and CS and outline techniques that are commonly used to measure both factors (Section “Introduction”). We intend this to provide the reader with relevant context for interpreting studies that are discussed later in the review. The concepts of CS and presence are discussed below (see Sections “Presence,” and “Cybersickness”), but in brief, these are complex phenomena with a multitude of individual differences (e.g., sex, gaming experience) and external factors (e.g., control of navigation, visual display parameters) thought to influence their occurrence. Each factor has been targeted using several measurement techniques, all of which vary with respect to how the measured variable is operationalized.

The second part of this review highlights studies that have directly measured the link between presence and CS, which we identified through a scoping literature search (Section “Evidence of a Presence-Cybersickness Relationship”). Given the large and historic importance of understanding and improving the issues of presence and CS, numerous studies have measured both factors and have even identified relationships between them. However, while approaches to solving the problems of presence and CS in VR have been tackled separately in large numbers of recent research papers and reviews, evidence of a possible link between them has seen very little discussion, particularly in recent years. VR has developed rapidly since its first implementation in the 1960s, and as such, early reports on the link between presence and CS may not apply to the current state of VR.

The third part of the review constitutes a broad overview of the associations between presence, CS, and other variables, since a large number of contradictory findings have been reported in the literature. These confounding factors emerge in part due to the rapid rise of VR, the multifactorial nature of both CS and presence, and the influence of other modulating factors such as sensory mismatch, display factors, and personal characteristics (Section “Associations with Other Variables”).

In our final section, and throughout the article, we aim to provide a synthesis of the research, with a special focus on unifying the discrepant findings about the nature of the presence-CS association. Our conclusions can be summarized as follows. First, there is more compelling evidence in support of a negative association between CS and presence than alternative relationships. The experimental results indicating a positive correlation between the two factors may be attributed to the necessity for settings to be “immersive” before CS can emerge. Second, there is considerable evidence for the role of sensory mismatch in both presence and CS. We also discuss the likelihood that sensory mismatch modulates a variety of factors that have been empirically linked with presence and CS (e.g., navigation control, display factors, vection). The strength of our conclusion is tempered by a need for additional high-powered studies in future research.

Our objective with this review is to provide an answer to the following question: What is the relationship between presence and CS in VR? Note that this review does not constitute a review of CS (see LaViola, 2000; Davis et al., 2014; Rebenitsch and Owen, 2016) or of presence (see Lombard and Ditton, 1997; Schuemie et al., 2001; Sadowski and Stanney, 2002; Biocca et al., 2003; Lee, 2004; Sanchez-Vives and Slater, 2005; for a meta-analysis see Cummings and Bailenson, 2016), but rather, of their interrelation. We address this relationship in order to answer pertinent questions in VR research: Does improving presence come at the cost of increasing CS, or can an intervention be conceived that improves presence and reduces CS? The second objective of this review is to provide a condensed view of the field of presence-CS research which we hope will prove useful to the next wave of studies on this complex relationship. While the majority of this review is focused on findings obtained in the disciplines of cognitive neuroscience and experimental psychology, the conclusions of the review are naturally relevant to human–computer interaction and human factors research.

### Presence

For over 40 years, the goal of achieving presence has been regarded as a defining aspect of a successful VR experience (Minsky, 1980). Although multiple definitions and dimensions of presence have been proposed (Sadowski and Stanney, 2002), the concept is almost universally described as the observer’s sense of psychologically leaving their real location and feeling as if transported to a virtual environment. Put simply, presence is the illusion of “being there” (Heeter, 1992). A variety of factors influence the likelihood that a user feels presence in a virtual environment (see Section “Associations With Other Variables”). For instance, the earliest VR implementations were built with an understanding that presence depends upon receiving correlated multisensory inputs that convey a simulated environment (the *cybernetic* approach to VR; Minsky, 1980; Herbelin et al., 2015). Many consider presence to be associated with the degree of environmental interaction (Slater and Usoh, 1993) as well as the fidelity and realism of information about the simulated landscape that is conveyed to the sensory modalities (Witmer and Singer, 1994). Individual differences in susceptibility to presence also play a large role (Witmer and Singer, 1998). Distinctions have been made between types of presence: *Physical presence*, the sense of physical relocation of the observer (IJsselsteijn et al., 2000); and *social presence*, the sense of being collocated with virtual agents (Heeter, 1992; Lombard and Ditton, 1997; Biocca et al., 2003). Several researchers have noted important distinctions between presence – the feeling of “being there” – and related concepts, such as *engrossment* and *immersion*. An individual may be highly attentive to a task in VR (engrossed) without feeling presence; similarly, the degree to which an individual is shut-off from the real world by a VR system (immersion) may not determine presence (Barfield et al., 1995; Slater et al., 1996; Nichols et al., 2000). Others have emphasized that presence is strongly modulated by perception of motor affordances of objects in VR (Dalgarno and Lee, 2010; Triberti and Riva, 2016), in addition to the importance of embodying a plausible virtual avatar in encouraging the sense that a virtual space is “real” rather than artifice (Slater et al., 2009; Grabarczyk and Pokropski, 2016). The embodiment of an avatar is in turn dependent on the synchronicity of sensory stimuli obtained by a VR user (Kilteni et al., 2012). At the same time, individual differences appear to modulate presence in response to VR content: Individuals who strongly express personality traits of openness, neuroticism, absorption, and extraversion tend to report higher levels of presence (Sacau et al., 2008; Weibel et al., 2010). The reason for this difference is unclear, although it is possible that the finding indicates a bias at the response level, rather than reflecting differences in the qualitative experience of presence across personality types.

#### Measurements of Presence

A wide range of methods have been used to measure presence in virtual environments. Although it has been argued that the subjective quality of presence necessitates a subjective measure such as verbal self-reports about the sense of “being there” (Sheridan, 1992), research has increasingly emphasized the need to measure the similarity between behavioral responses in the real world and in a mediated environment as an objective index of presence (Bailenson et al., 2004; Slater, 2004a; van Baren and IJsselsteijn, 2004). As such, presence measures are often classified as *subjective* or *objective* measures, which can be further broken down into subcategories.

Objective measures include biomarkers which might relate to presence (e.g., obtained from heart rate and skin conductivity), behavioral measures (e.g., reflexive responses to dangerous stimuli, postural sway in response to visual stimulation), or measurements related to task performance in the virtual environment. van Baren and IJsselsteijn (2004) provide a detailed list of examples that employ each category of measurement tool. However, several other techniques for measuring presence have been studied in recent years, including neuroimaging (functional magnetic resonance imaging, fMRI; Baumgartner et al., 2008; Clemente et al., 2013) and electroencephalogram (EEG; e.g., Baumgartner et al., 2006; Clemente et al., 2014) which show potential for identifying neural correlates of presence in VR. The search for objective markers of presence is particularly pressing, given that established presence questionnaires have been criticized for several limitations, including a lack of including an inability to quantitatively discriminate between otherwise clearly distinguishable virtual and real life experiences (Usoh et al., 2000), measuring the post-exposure memory of presence rather than presence itself (Usoh et al., 1999), and a lack of sensitivity to presence compared with behavioral measures (Bailenson et al., 2004; Slater, 2004a).

Subjective measures are obtained either through questionnaires administered following VR exposure; self-report ratings solicited during VR exposure; verbal or written reports of the qualitative experience of presence; or psychophysical magnitude estimation/matching paradigms. Despite their criticisms, questionnaires have been the most common approach to measuring presence. The dominant *multi-item* scales include the “Presence Questionnaire” (*PQ*; Witmer and Singer, 1998), a 32-item list of seven-point questions that are used to generate scores on subscales such as *realism, possibility to act*, and *quality of interface*. The same authors developed the Immersive Tendencies Questionnaire (ITQ; Witmer and Singer, 1998) as an index of an individual’s likelihood of feeling immersed in virtual settings. The 29 items of the ITQ relate to the tendency to become involved in activities, to maintain focus in activities, and the tendency to play video games. As such, the ITQ can be taken as an index of “trait” tendency toward feeling (or reporting) presence. Other common scales include the Igroup Presence Questionnaire (IPQ; Schubert, 2003), which was developed to measure *spatial presence, involvement*, and *realness* of a simulated experience, and comprises a list of 14 items. A popular scale developed by Slater, Usoh, and Steed (SUS; Usoh et al., 2000) consists of a brief, 6-item questionnaire that generates a single score to convey how “present” the user felt in the virtual setting. Many other scales have been employed, including Likert-type rating scales, analog (continuous) ratings, or *single-item* measures (e.g., “To what extent did you feel present in the environment, as if you were really there?” Bouchard et al., 2004).

Each variety of measurement scale is accompanied by benefits and shortcomings. While multi-item presence questionnaires can provide a detailed assessment of the multiple dimensions that may underlie presence, the appeal of single-item scales is in their un-intrusive and rapid assessment of presence (Bouchard et al., 2004). Single-item scales may also be less prone to memory deterioration following virtual environment exposure, and can be administered several times during exposure to VR (although it should be noted that repeat probing of a VR user may interrupt and diminish the experience of presence). When compared with lengthy questionnaires, single item scales are potentially more accessible to some participants, including children or individuals with learning difficulties. A thorough discussion of the limitations and utility of each type of scale is provided by van Baren and IJsselsteijn (2004).

### Cybersickness

As with presence, several definitions have been proposed for what we term here CS. We follow the definition outlined by Stanney et al. (1997): CS is a constellation of symptoms of discomfort and malaise produced by VR exposure. CS is typically categorized as a form of visually induced motion sickness (VIMS), which describes any sickness produced by observation of visual motion, and it is distinct – but symptomatically similar to – simulator sickness (SS), which is produced by vehicle simulators. A slight distinction has been made between the experience of CS and SS; while CS is characterized by a prevalence of disorientation symptoms, SS appears to be predominated by oculomotor symptoms (Stanney et al., 1997). While many individuals experience CS in VR, others appear to be robust to the symptoms. Causal factors have been identified and discussed in great detail, including mismatches between observed and expected sensory signals (Reason and Brand, 1975; Rebenitsch and Owen, 2016), self-motion (McCauley and Sharkey, 1992), visual display characteristics (Moss and Muth, 2011), and gameplay experience (Knight and Arns, 2006; Gamito et al., 2008).

#### Measurements of Cybersickness

As with presence, the magnitude of CS experienced by a VR user has been estimated using both objective and subjective measures. Objective measures may involve analysis of physiological markers. Increases in bradygastric activity, respiration rate (Kim et al., 2005; Dennison et al., 2016) heart rate (Nalivaiko et al., 2015), and skin conductance at the forehead (Golding, 1992; Gavgani et al., 2017) provide robust measures of CS. Behavioral signs such as early termination of a VR experience (Kinsella, 2014) and task competence (Lin et al., 2015; Nalivaiko et al., 2015) also indicate the extent to which an individual experiences CS.

The most common approach to assessing CS involves subjective measures, particularly multi-item questionnaires such as the Simulator Sickness Questionnaire (SSQ; Kennedy et al., 1993) which includes 16 items (e.g., *eyestrain, dizziness*, and *headache*) on a four-point scale (*none, slight, moderate*, or *severe*). Common practice with the SSQ is to generate a *total* sickness score as well as scores for each subscale of *oculomotor discomfort, disorientation*, and *nausea*. A shortened version of the SSQ (Short Symptoms Checklist, SSC; Cobb et al., 1995), consisting of two items from each subscale, has been developed and employed in a small number of studies (Wilson et al., 1997; Cobb et al., 1999). Given the dynamic nature of CS, which tends to increase during VR exposure and slowly dissipate following VR termination, there are clear challenges involved in using one-shot questionnaire measurements of CS. Single item scales for measuring CS have also been developed and validated, providing an efficient method for assessing the temporal evolution of CS (e.g., Fast Motion Sickness Scale; Keshavarz and Hecht, 2011b). The near future of CS research will likely involve an integrated approach, where physiological assessments (see Section “Physiological Measures”) are combined with multi-item and single-item questionnaires that are completed both during and after VR exposure.

### Shared Measurements of Presence and Cybersickness

Multiple measurement techniques are common to both presence and CS. These can be broadly categorized as physiological markers (e.g., recordings of neural or dermal activity), or task-performance based measures (e.g., reaction times, performance accuracy). Here we describe these approaches to measuring both factors, and discuss how the overlap between measurement approaches causes difficulty with interpreting the true relatedness of the factors.

#### Physiological Measures

Physiological methods have been applied to the measurement of both presence and CS, which presents a significant potential problem in understanding how the two factors are related. Indices of autonomic nervous system activity offer reliable measures of the stress response, and this stress/alarm response is linked to both presence and CS. Physiological correlates of acute CS symptomatology (sweating, nausea, skin pallor, and increased heart rate) reflect the neuroendocrine stress response (Harm, 2002; Kim et al., 2005; Ohyama et al., 2007). Equally, the magnitude of a stress response to a virtual environment is often considered an indicator of presence (Bouchard et al., 2008; Ling et al., 2013). Research on presence in stressful environments (such as standing at the top of a height) suggests that assigning a personal relevance to the environment due to presence (e.g., “I could really fall into this pit”) leads to heightened physiological reactions such as increased heart rate and skin conductance (Meehan et al., 2001, 2003; Wiederhold et al., 2001; Zimmons and Panter, 2003) This physiological response is thought to be caused by the release of adrenocorticotropin hormone (ACTH), growth hormone, and other hormones by the pituitary gland (Harm, 2002). We are unaware of any studies that assess hormonal correlates of presence, although the neuroendocrine response to motion sickness has been studied extensively. Evidence from physiology indicates that the secretion rate of ACTH and vasopressin in response to a visual motion stimulus is correlated with susceptibility to motion sickness (Eversmann et al., 1978; Kohl, 1985; Kim et al., 1997). In support of this physiological basis, Asian individuals are more susceptible to motion sickness, which may be related to the increased levels of vasopressin release observed in this population (Stern et al., 1996; Klosterhalfen et al., 2005). Neurophysiology studies have also produced an advanced understanding of the brain mechanisms underlying motion sickness. The emetic component of motion sickness is thought to be controlled by a pathway that involves the vestibular nuclei in the brainstem (Yates et al., 1998). These nuclei, which produce emesis when externally stimulated (Miller and Wilson, 1983), show modulated activity in response to levels of hormones and neurotransmitters such as GABA, dopamine, and ACTH (Balaban et al., 1989). A primary function of the vestibular nuclei is to project information about self-motion to the thalamus and vestibular cortex (Glover, 2009), and it has been claimed that incongruent sources of self-motion information that are integrated here significantly contribute to CS (Yates et al., 1998; Oman and Cullen, 2014; further discussion can be found at Section “Sensory Mismatch”). On the other hand, our understanding of the neural mechanisms for feeling presence are much weaker; understandably so, given the much more qualitative and phenomenological nature of presence. While there is some evidence from EEG and fMRI recordings that presence is associated with increased parietal and prefrontal cortex activation (Baumgartner et al., 2006, 2008), this field of research will likely grow rapidly given the recent increase in prevalence of VR technology.

#### Task Performance

Feeling present in a virtual space appears to enhance task performance. It has been shown that feeling presence is related to improved performance in the game of chess in VR (Slater et al., 1996), human interaction (Stanney et al., 2002), engine maintenance tasks (Cooper et al., 2016), and simple psychomotor tasks (Witmer and Singer, 1994; but c.f. Singer et al., 1995). In one study, a striking 95% of variability in presence ratings (PQ) was explained by variance in time to completion of an engineering task (Cooper et al., 2016). The conceptual link between presence and task performance appears weak, however (van Baren and IJsselsteijn, 2004), and it is possible that the relationship between presence and task performance measures is strongly mediated by other factors, such as experimental instructions, individual motivation, and even CS.

The inverse correlation between CS and task performance is well-supported, with several studies showing that symptoms of sickness are linked to decreased task performance (Frank et al., 1988; Kennedy and Fowlkes, 1992; Kennedy et al., 1993; Lerman et al., 1993; Nelson et al., 2000; Stanney et al., 2002; Kim et al., 2005). Ultimately, many who suffer from CS elect to terminate a session of VR early and therefore cannot complete the given task (DiZio and Lackner, 1997). In studies where no relationship between task performance and sickness severity is found, it is often claimed that symptoms were too mild to interfere with task performance (Nelson et al., 2000; Bos et al., 2005).

Using task performance to measure both CS and presence leads to some obvious problems in interpreting their relatedness. The evidence suggests that task performance is more indicative of CS than presence, although a conservative approach should be considered: Since task performance measures are likely to conflate multiple constructs, they are not ideal for use in isolation and should be used in conjunction with other metrics. Note that this caution applies equally to measures such as “enjoyment” as indices of presence or CS (e.g., Wilson et al., 1997; Slater, 2004b; Waterworth et al., 2015).

## Evidence of a Presence-Cybersickness Relationship

**FIGURE 1:**
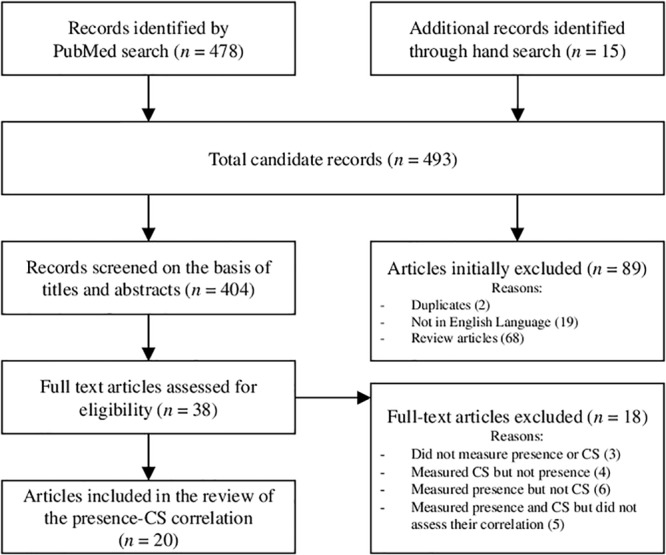
PRISMA flowchart indicating the method for identifying and selecting articles that depict the relationship between presence and CS. Based on Moher et al. (2009).

**FIGURE 2:**
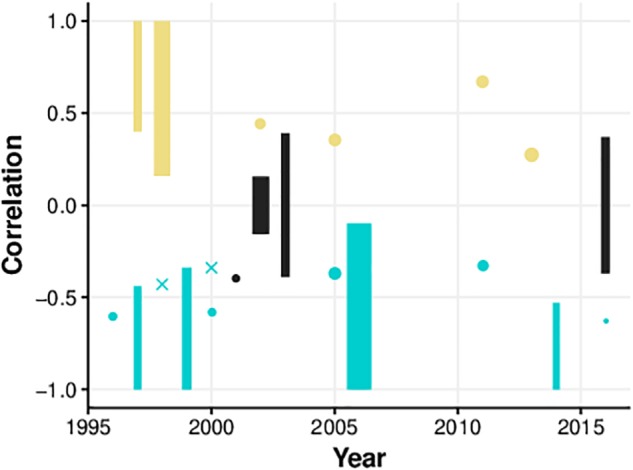
Correlations by year of publication for experiments reported in Table 1. Width of elements reflects degrees of freedom (maximum = 385, minimum = 6). Yellow indicates positive correlations, cyan indicates negative correlations, and black indicates null correlations. Since some studies did not report correlation values, vertical bars are used to indicate the range of possible Pearson *r* correlation values given the reported sample size. Crosses indicate that degrees of freedom were not reported.

There are a number of documented efforts to record presence and CS concurrently. Within this literature there is significant disagreement with respect to the strength and direction of the relationship. Here we outline a literature search of studies that report positive, negative, or null correlations between presence and CS, obtained using a structured literature search (Figure 1). We report the display device used, the task, the sample size, and statistics for each effect (if reported) in a summary table (Table 1) and an illustration (Figure 2). Finally, we identify where the more convincing evidence appears to lie, and discuss some possible reasons for the discordance in findings.

**Table 1:** Studies assessing the presence-CS link: Negative, positive, and null correlations.

Study	VR Task	Device	*N*	Sign	Statistics	Measures
Witmer et al., 1996	Office navigation	Fakespace Labs BOOM2C	22	–	*r*(20) = –0.60	Presence: PQ CS: SSQ
Wilson et al., 1997 (1)	n.r.	Division dVisor	20	–	n.r.	Presence: PQ CS: SSC
Witmer and Singer, 1998	Multiple tasks	n.r.	n.r.	–	*r* = –0.43 across 4 exp.	Presence: PQ CS: SSQ
Usoh et al., 1999	Room navigation	Virtual Research V8	33	–	n.r.	Presence: SUS CS: SSQ
Nichols et al., 2000	House navigation	Division dVisor	20	–	*r*(18) = –0.58	Presence: SUS CS: SSQ
Stanney, 2000, Unpublished	Maze navigation	n.r.	n.r.	–	*r* = –0.34 (d.f. n.r.)	n.r.
Kim et al., 2005 (1)	Town navigation	Projection screen	61	–	*r*(59) = –0.37	Presence: PQ CS: SSQ
Knight and Arns, 2006	n.r.	Projection screen	387	–	n.r.	Presence: Likert CS: SSQ
Busscher et al., 2011	Video observation	Cybermind Visette Pro	43	–	*r*(41) = –0.33	Presence: IPQ CS: SSQ
Milleville-Pennel and Charron, 2015	Driving simulation	3 LCD screen surround	14	–	n.r.	Presence: Authors’ own scale CS: SSQ
Cooper et al., 2016	Car wheel change	Projection screen	8	–	*r*(6) = –0.63	Presence: PQ CS: SSQ
Wilson et al., 1997 (2)	Duck shooting	Virtual I/O i-glasses	24	+	n.r.	Presence: Startle, subjective score CS: SSC
Bangay and Preston, 1998	Rollercoaster	HMD (model not reported)	143	+	n.r.	Presence: Subjective score CS: SSQ
Lin et al., 2002	Driving simulation	Projection screen, CrystalEyes glasses	40	+	*r*(38) = 0.44	Presence: SUS (modified) CS: SSQ
Kim et al., 2005 (2)	Town navigation	3D Visual and Auditory Environment Generator	61	+	*r*(59) = 0.35	Presence: ITQ CS: SSQ
Liu and Uang, 2011	Grocery shopping	n.r.	60	+	*r*(58) = 0.67	Presence: PQ CS: SSQ
Ling et al., 2013	Public speaking	eMagin Z800 3DVisor	88	+	*r*(86) = 0.28 (ITQ/SSQ)	Presence: ITQ CS: SSQ
Mania and Chalmers, 2001	Listening to a seminar	Prototype HP HMD	54	×	*r*(16) = –0.4	Presence: SUS (modified) CS: SSQ
Seay et al., 2002	Driving simulation	Projection screen	156	×	n.r.	Presence: PQ CS: SSQ
Robillard et al., 2003	Asked to approach phobogenic stimuli (spiders)	I-Glass HMD	26	×	n.r.	Presence: PQ, ITQ, subjective score CS: SSQ
Ryan and Griffin, 2016	Sitting in a café	Oculus Rift DK2	28	×	n.r.	n.r.

*+ = positive. – = negative. × = null correlation. n.r. = not reported. SSQ = Simulator Sickness Questionnaire, Kennedy et al., 1993; SSC = Short Symptoms Checklist, Cobb et al., 1995; SUS = Slater-Usoh-Steed Questionnaire, Usoh et al., 2000; ITQ = Immersive Tendencies Questionnaire, Witmer and Singer, 1998; IPQ = Igroup Presence Questionnaire, Schubert, 2003; PQ = Presence Questionnaire, Witmer and Singer, 1998.*

### Review Method and Results

Our general method followed The PRISMA Statement (Moher et al., 2009), which provides a standardized set of items for reporting in systematic reviews. The primary aim of our review was to identify research studies that directly examine the relationship between presence and CS. Our criterion for inclusion was that the studies must have measured both presence and CS produced by the use of VR and analyzed the correlation between the factors. The method we used was to conduct a database search on PubMed, PsycINFO, and Google Scholar for publications that conducted experimental studies with VR (search term: *virtual reality*), including terms related to CS (*cybersickness, nausea, sickness*, or *emetic*), and terms related to presence (*presence, immersion, immersiveness*, or *telepresence*). Initially, there were 478 results returned. Figure 1 depicts the procedure for identifying and selecting records from the literature search.

As demonstrated by Figure 1, significant attrition occurred in the article selection process. We read the abstracts of all the papers and found that the vast majority of the results (∼366 of 404 records screened) referred to presence and CS briefly with regard to their relevance to the advancement of VR in rehabilitation, education, or consumer settings, or they used the search terms in a general sense. Several results containing instances of the key terms “presence” or “immersion” were unrelated to the sense of “being there” (e.g., “Cybersickness in the presence of scene rotational movements along different axes”; “immersion in VR” used as a synonym for “exposure to VR”; numerous other examples can be seen by the reader upon reproducing the search results). Terms such as “presence” are highly context-specific, and several studies not contained in the results use terms for CS that are general and difficult to identify with a literature review search, such as “negative effects.” Another portion of the search results (18 records) measured only presence, or only CS, or measured neither. Many of these studies focused on the effect of an experimental manipulation on presence, where CS measures were collected solely in order to confirm that CS was low or negligible and was unaffected by the manipulation.

Table 1 provides an overview of each study identified using our search, including details of the VR task included in the experiment, the device used to depict the virtual environment, and the scales or measures used to acquire data on CS and presence. The table also includes the sample size and statistics for the relevant correlations. In numerous cases these details have not been reported by the study authors. Nonetheless, the details of the 20 publications that have directly measured the correlation between presence and CS may prove informative for future studies on human factors in VR.

### Summary of Studies Identified by the Literature Search

We describe the studies that we identified with the literature search below. We also describe the original authors’ conclusion about the nature of the presence-CS association based on their findings, where this information was available. Following this summary, we outline our interpretation of how presence and CS are related based on a synthesis of the literature that we reviewed here (see Section “Conclusion: How Are Presence and Cybersickness Related?”).

#### Studies Reporting a Negative Correlation

Reports of negative correlations between presence and CS were reported early by Witmer et al. (1996) and Witmer and Singer (1998). Data from Witmer et al. (1996) showed a large negative correlation between scores on a presence questionnaire and self-reported symptom severity on a CS scale. The authors proposed that participants who experience symptoms are more internally focused and less able to process features of the environment, thus limiting the sense of presence.

Witmer and Singer (1998) reported data obtained in four experiments that helped to establish the Presence Questionnaire (PQ) and its relationship to CS. The significant reported correlation was taken as evidence that experiencing symptoms of CS tends to diminish the feeling of presence via distraction or a reduction in the user’s involvement in the virtual environment.

In a study carried out by Wilson et al. (1997), a negative relationship was observed between the interface quality subscale of the PQ and scores on the SSC scale in VR. The authors proposed that sickness symptoms may detract from presence, or that presence reduces the awareness of sickness symptoms. Evidence supporting this finding was gathered by Usoh et al. (1999) using a virtual room navigation task. Here, the oculomotor subscale of the CS scale used in this experiment was higher when presence scores were low, suggesting that oculomotor discomfort might have produced an internal focus in users.

Nichols et al. (2000) found evidence for a negative correlation between presence and CS during virtual house exploration. The task required several basic object manipulations (e.g., picking up and placing objects) using a three-dimensional mouse. A negative association between total CS ratings and presence scores was obtained following exposure to the virtual environment. The authors suggested that individuals with more symptoms of sickness may have concentrated less on the task, and may have been more attuned to the deficiencies of the virtual environment simulation (e.g., low refresh rate).

A negative relationship between subjective ratings of presence and sickness severity was obtained by Stanney (2000, Unpublished); reported informally by Stanney (2002). A negative correlation of a similar magnitude was obtained during virtual town navigation by Kim et al. (2005), who showed that CS and presence (particularly the feeling of “control” in the VR environment) were negatively related. Unfortunately, while the same authors also obtained physiological signals (e.g., heart rate, EEG), they did not report the full set of possible correlations between physiological data, CS scores, and presence ratings.

A brief report of a large-sample study by Knight and Arns (2006) supported the existence of an inverse relation between presence and CS in immersive VR. The results showed significant chi-squared tests indicating that total SSQ scores decreased with increasing levels of presence. Knight and Arns (2006) also collected data on several other related factors, such as previous game play experience, motion sickness susceptibility, and participant sex, which permits inferences about latent causes for both presence and CS, although not all correlations between measures were reported (e.g., despite collecting gameplay experience, it was not specified if this factor was correlated with presence as in other studies; see Section “Gaming Experience”).

Busscher et al. (2011) measured CS and presence while participants watched a video on a television in a simulated lounge environment. The authors described a significant negative correlation between the two factors and took this correlation as evidence that maximizing presence in VR leads to a suppression of CS, which was taken as evidence that future interventions may be able to tackle both issues concurrently.

A study using a partial least-squares regression method identified an inverse association between presence and CS in a driving simulation task (Milleville-Pennel and Charron, 2015). The authors collected several possible predictors of CS including driving experience, tendency toward frustration, and presence, and found that presence loaded negatively on a latent variable that was termed “pre-disposition to sickness.” Milleville-Pennel and Charron also validated the single-factor construction of the SSQ and confirmed that the sub-components of the SSQ (nausea, oculomotor discomfort, and disorientation) each contribute approximately one third of the variance in overall levels of CS. This is an important finding given the high prevalence of SSQ use in studies of CS.

A recent study from Cooper et al. (2016) showed that subjective presence ratings were negatively associated with discomfort ratings that were collected following an immersive “pit stop” scenario. Although sample size was small (*N* = 8), the authors took the evidence as support for the utility of a multisensory cueing approach to improve presence and reduce the severity of sickness in VR.

#### Studies Reporting a Positive Correlation

As described above, Wilson et al. (1997) identified a negative correlation between presence and symptoms of sickness following the use of VR in one experiment, but in a second experiment, despite the fact that the same scales were used to measure the two factors, found evidence for the positive relationship. Participants conducted a virtual “duck shooting” task and completed a CS checklist, while behavioral (startle response) and subjective ratings of presence (presence questionnaires and awareness of background music manipulation) were collected.

Liu and Uang (2011) identified a positive relationship between presence and CS was in a virtual shopping task. Older adult participants were asked to search for specific items on shelves in a virtual grocery store. Results indicated a strong positive correlation between presence and CS, and the authors suggested an increase in presence causes CS to increase. An in-depth interpretation of the study is limited due to the fact that the authors did not specify certain details, such as the duration of VR exposure or the display device used.

Ling et al. (2013) report finding a positive link between CS and scores on the immersive tendency questionnaire (ITQ) that was administered after participants completed an anxiety-inducing task in VR. This was taken as evidence that individuals who experience more presence also experience more CS, and this conclusion was supported by evidence of a positive correlation between ITQ scores and levels of spatial presence. Despite these associations, there was no correlation found between spatial presence and CS. The authors suggested that the expected relationship between spatial presence and CS did not emerge due to the high cognitive demand of the public speaking task that may have modulated presence and CS in different ways.

Lin et al. (2002) obtained a strong positive correlation between presence and CS ratings in a CAVE-like driving simulator, from a sample of 10 participants. The authors state that there was a low level of interactivity in their VR task compared to other similar studies (e.g., Nichols et al., 2000, who found the opposite relationship), and noted that the level of interactivity afforded in virtual environments is likely to alter the relationship between presence and the severity of sickness.

As described above, Kim et al. (2005) identified a negative correlation between CS and presence (“user control” factor of the PQ). In the same study, the authors found that the direction of the relationship depended upon the questionnaire that was used: A positive correlation was documented between CS and the Involvement factor of the ITQ. This divergence was not discussed by the authors. This finding highlights one of the problems involved in characterizing presence, given the discrepancy between trait (ITQ involvement) and state (PQ control) measures of the phenomenon.

Slater et al. (1996) speculated that vection in VR was a common contributing factor to both CS and presence, stating that a positive correlation between presence and CS would therefore be “not surprising.” Indeed, some of the more convincing (albeit, indirect) evidence of a positive CS-presence relationship has emerged from vection research. Hettinger et al. (1990) reported that a vection-inducing stimulus can produce VIMS, and more recently, Keshavarz et al. (2014) have shown that even “auditory vection” (i.e., vection produced by an auditory self-motion cue) can produce sickness symptoms. Other links between vection and VIMS have been discussed in a recent review (Keshavarz et al., 2015). Taken together with evidence of a strong relationship between vection and presence, it seems logical that increases in the sense of vection in a VE should improve presence, and also cause CS to increase. However, evidence on such a link is unclear (see Section “Vection”).

#### Studies Reporting a Null Correlation

Some studies have reported null correlations between presence and CS. These reports are very sparse, possibly due to a bias for significant results (e.g., Open Science Collaboration, 2012, 2015). Mania and Chalmers (2001) report a study where participants were asked to observe a video in VR and to report their level of presence and CS. The authors found no significant relationship between presence and CS, perhaps because CS scores were quite low across participants, although a trend toward a negative correlation was observed.

In an investigation by Seay et al. (2002), a large sample of participants conducted a driving simulation task and reported their level of presence and CS. Results indicated no correlation between presence and any subscales of the CS measure. However, the same authors found main effects of an experimental manipulation – field-of-view angle, 180° vs. 60° – on both presence and the nausea subscale of their CS measure. In light of these inconsistent results, the authors concluded that factors such as field-of-view can prove to be a “double edged sword,” increasing presence but also increasing sickness. Similarly, Bangay and Preston (1998) did not analyze whether a correlation existed between their measures of CS and presence, but identified that those who experienced CS were likely to report high levels of immersion in the VR environment.

A recent study found a null correlation between presence and CS while participants observed an animated avatar in a virtual café using a head-mounted display (Ryan and Griffin, 2016). It is unclear how CS was measured in this study, and levels of CS were also reported to have been very low which may have limited the power of the analysis. It is notable that of the studies reviewed here, this study is the only one to have used a modern consumer-oriented VR device (Oculus Rift DK2). Since these devices have become extremely popular for VR research in recent times (Peer and Ponto, 2017), it is likely that studies on the presence-CS relationship in the coming years will use this device or a similar one, thus reducing much of the inter-experiment variability that is attributable to different display conditions.

### Conclusion: How Are Presence and Cybersickness Related?

The balance of evidence favors the interpretation that presence and CS are negatively related. There are several reasons for this. First, the number of research studies that report the existence of a negative correlation outweighs the number of studies that report the opposite. Studies that describe an inverse relationship also tend to provide more compelling results: Where studies have observed a positive correlation between presence and CS, the study often fails to confirm this relationship in another section of the same study (e.g., Wilson et al., 1997; Kim et al., 2005; Ling et al., 2013). In some of the studies cited above that identified a positive correlation, interpretation of the data is limited by the absence of important details (e.g., Liu and Uang, 2011, did not describe device; Wilson et al., 1997, did not report test statistics).

Although a positive correlation between presence and CS has been anticipated or assumed by some researchers (e.g., McCauley and Sharkey, 1992; Slater et al., 1996), it is likely that positive associations arise due to the fact that “immersiveness” is required in order for an individual to experience CS. Immersiveness here refers to the extent of sensory “submersion” experienced by a user with a given VR system, such that external sensory cues are obstructed (Biocca and Delaney, 1995); accordingly, desktop systems and head-mounted displays (HMD) are classified as low and high in immersiveness, respectively. Observing a bright, dynamic movie on a desktop monitor is a comfortable experience for most, but viewing the same scene in a VR headset can often produce CS. Similarly, the sense of presence is heightened by the use of immersive systems. As such, immersion in VR leads to the possibility of both CS and presence emerging.

What mechanism causes this inverse association between CS and presence? It has been claimed that the sense of presence suppresses CS, since attention is directed away from intrusive factors such as sensory conflict (e.g., Busscher et al., 2011; Cooper et al., 2016). Alternatively, the distracting effects of CS may suppress attention to the VR environment that is required for presence to occur (e.g., Wilson et al., 1997; Witmer and Singer, 1998; Usoh et al., 1999; Nichols et al., 2000). More than likely, both of these assertions are true; they are not mutually exclusive. The relationship is also clearly mediated by other factors that appear to affect CS and presence inversely. A large number of associated variables have been identified, and although there is insufficient research to construct a precise model of their contribution to either factor, research suggests crucial roles played by sensory mismatch, display factors, navigation control, sex, and other factors (for an in-depth discussion, see Section “Associations With Other Variables”).

There are also important limitations to several of the studies that reported negative presence-CS relationship, such as missing test statistics, or a failure to describe display device features. Many of the studies that reported a negative correlation were conducted before the advent of modern consumer-oriented VR technology, and their findings may not entirely replicate using current hardware devices. A major limitation of almost all studies described above is the small sample size used in the experiments. With one or two exceptions, the studies above on average wield very low statistical power for detecting medium effects. In the single case where an *a priori* power analysis was conducted, a desired power of 80%, which is on the low-end of “adequate” (Button et al., 2013), requires approximately 85 participants. An *a priori* power analysis was reported in only one of the studies described here (Ling et al., 2013), and we estimate that only two other studies described here were likely to have attained >80% statistical power: The brief report by Knight and Arns (2006), who measured a convenience sample of *N* = 387; and a conference paper by Seay et al. (2002), who measured a sample of *N* = 156. Evidently, there is a need for the adoption of a more scientifically rigorous approach toward statistical power, as has been reported widely across the fields of psychology and neuroscience (Open Science Collaboration, 2012, 2015; Button et al., 2013).

In Figure 2 we depict the correlation between CS and presence obtained in the studies that we reviewed and discussed here. On inspecting this figure, it is notable that very few recent studies have empirically examined the strength of the association between presence and CS. While recent literature often discusses both factors in the context of VR (e.g., Aardema et al., 2010; Terziman et al., 2010; Kober and Neuper, 2012; Serafin et al., 2016), they are often described only for the purpose of highlighting the nuisance of CS and the desirability of presence. For instance, presence and CS are both measured by Nolin et al. (2016), but the strength of correlation is not reported. Kober and Neuper (2012) stated that participants in their study of presence in VR also completed a CS questionnaire, but the authors only used these data to confirm that CS was at a low level overall. Similarly, Corrêa et al. (2017) assessed CS and presence, simply reporting that CS was low in the participants tested. Baus and Bouchard (2017) used high self-reported CS levels as an exclusion criterion, and did not assess the relationship with levels of presence. Kim K. et al. (2012) also report a study where CS and presence measures were both collected, and although their manipulation (visual display device: Desktop/HMD/CAVE) affected both CS and presence, their relationship is not reported. While the nature of the presence-CS link may not have been a major focus of any of these studies, since the data clearly existed, it is rather unfortunate that no analysis was reported. The presentation of these data in future studies where the data are collected would provide valuable information to developers and researchers interested in advancing the understanding of the human experience in VR. It should also be noted that a diverse variety of VR technology has been used in the studies reported above, spanning from older display devices (e.g., Division dVisor, Fakespace BOOM2C) to more recent consumer headsets (Oculus Rift DK2). Modern VR devices such as HTC Vive and Oculus Rift CV1 are more similar to one another with respect to many characteristics (field-of-view, refresh rate, tracking latency) than were older systems (Peer and Ponto, 2017). Another limitation of the existing literature is a severe underreporting of the input techniques adopted for environmental navigation and interaction. In the coming years, the consistency of findings in the field of human factors in VR will likely benefit from a natural standardization of display tools.

## Associations With Other Variables

As research has investigated the nature of the relationship between presence and CS, a variety of candidates for mediation of the relationship have emerged. Although no studies have attempted to estimate the magnitude of the contribution of each of these factors, here, we present a synthesis of the literature that offers clues as to the most important mediators on the presence-CS relationship. We make connections between these sometimes disparate studies, and highlight the interactions between some of the factors associated with both presence and CS.

### Sensory Mismatch

Sensory cues gathered from multiple channels (proprioception, vision, vestibular, etc.) are used to perform continuous updating about the estimated state of the world and of the body (Calvert et al., 2004). Therefore, when simulating a virtual environment, congruence between the information that is obtained and that which is expected (either because of prior experience, or because of expected correlations with another sensory channel) plays a large role in the experience.

The understanding of how sensory mismatch contributes to CS and presence has historically been limited due to the challenge of directly manipulating or measuring sensory conflict in experimental settings (Riccio and Stoffregen, 1991; Oman and Cullen, 2014). For instance, only recently have convincing results emerged that are consistent with a neural signature for sensory mismatch (e.g., Brooks and Cullen, 2013, 2014). Nonetheless, theoretical accounts have highlighted the role played by the congruence of sensory cues in both presence (Slater et al., 1995; Bowman and McMahan, 2007; Henderson et al., 2007) and CS (Reason and Brand, 1975; Oman, 1991; Rebenitsch and Owen, 2016). It is clear that the addition of high-fidelity, multimodally congruent information is beneficial to an increase in presence. Participants in a VR navigation task show increased presence when binaural auditory information is presented compared to vision-alone conditions (Larsson et al., 2002). Introducing multisensory feedback cues (tactile, auditory, and visual) in a manual VR task also enhances presence (Cooper et al., 2016; also see Hecht et al., 2006). Viaud-Delmon et al. (2006) demonstrated that adding auditory cues to a virtual environment (i.e., enhancing the immersiveness of the simulation) increases presence, but also leads to a rise in levels of CS.

However, to our knowledge, there is little research on the disruption of presence when multimodal cues are in conflict. The most relevant literature in that context relates to vection, a strong correlate of presence (Riecke, 2010). The evidence of a relationship between vection and sensory mismatch is inconsistent: Visual-vestibular cue mismatch has been linked to a decreased sense of vection (Wong and Frost, 1981; Weech and Troje, 2017) or to enhanced vection (Kim J. et al., 2012; Palmisano et al., 2012; also see Section “Vection”). Future research will be needed to establish causality between cue conflict and presence, perhaps by assessing the tendency for breaks in presence when multimodal cues are put experimentally into conflict.

It has been theorized that CS in VR is produced as a result of mismatches in information across sensory streams, or conflicts between observed and expected sensory cues, particularly with respect to visual-vestibular cue conflict (Reason and Brand, 1975; Oman, 1991; Rebenitsch and Owen, 2016). The link between multimodal cue mismatch and the symptoms of CS has been attributed to the detection of sensory dysfunction (Treisman, 1977). Motivated by these theoretical accounts, several researchers have attempted to prevent CS through a sensory conflict reduction approach, with some successful results (e.g., Reed-Jones et al., 2007; Cevette et al., 2012; Gálvez-García et al., 2015; Zao et al., 2016). This research has produced evidence that CS symptoms are reduced when sensory stimulation is used to “recouple” multimodal streams of information in VR. Visual-vestibular cue mismatch is a particular source of discomfort, and this manner of conflict is extremely common in VR applications (LaViola, 2000; Rebenitsch and Owen, 2016). By reducing this mismatch using vestibular stimulation, CS appears to be mitigated (Reed-Jones et al., 2007; Cevette et al., 2012; Gálvez-García et al., 2015). Other research has adopted a sensory reweighting approach to encourage conflicting cues to be quickly disregarded using “noisy” vestibular stimulation, rather than “recoupling” the sensory streams (Weech et al., 2018a). Taken together with work showing that vection is also facilitated when noise is applied to the vestibular sense (Weech and Troje, 2017), the sensory reweighting approach appears to be a promising method for maximizing presence and minimizing CS. However, further refinements of the sensory stimulation methods currently used will be vital before a viable practical application can be achieved.

### Display Factors

Reports show that visual display characteristics such as frame rate and field-of-view influence both presence and CS. Higher frame rates are associated with higher self-reported presence ratings due to the increased realism afforded by smooth motion (Meehan et al., 2001). Low visual display frame rate (<20 fps) has long been known to generate motion sickness in simulated environments (e.g., Jones et al., 2004), leading to a focus on high, stable refresh rates in best practice guides for VR development (Oculus, 2017). This guide also emphasizes that latencies between observer motion and visual self-motion feedback should be minimized in order to avoid generating nausea, although the link between motion-to-photon latency and CS has been disputed (Meehan et al., 2003). Higher field-of-view also leads to increases in presence (Prothero and Hoffman, 1995), but at the same time, field-of-view increases lead to higher CS (Lin et al., 2002). It was suggested by Lin et al. (2002) that the effect of field-of-view on both factors might be mediated by its effect on illusory self-motion perception (vection) produced by large-field visual motion.

Evidence suggests that stereoscopy influences both presence and CS. Research has identified links between stereopsis and CS, likely due to the accommodation-vergence conflict introduced by stereoscopic 3D displays. For instance, viewing stimuli on certain 3D displays can increase measures of visual discomfort that are characteristic of CS, such as eyestrain (Emoto et al., 2004; Pölönen et al., 2009; Lambooij et al., 2011). As well, VIMS has a strong relationship to stereoscopy: Observing a simulated roller-coaster stereoscopically leads to increased VIMS symptoms compared to monocular viewing (Keshavarz and Hecht, 2012). Stereoscopic viewing of a virtual environment also takes advantage of the learned relationship between binocular disparity and object depth, increasing the naturalness of the viewing experience. Ling et al. (2013) show that providing stereoscopic cues appears to enhance presence (SUS; effect size Cohen’s *d* = 0.24) and spatial presence (IPQ; *d* = 0.29 in a public speaking task. Although the authors also predicted a relationship between stereoscopic acuity and presence, no evidence of such a link was observed. The authors suggested the link between presence and stereoscopy may be even stronger than implied by their results, given that their public speaking task involved very little binocular disparity. Indeed, a stronger link was observed by IJsselsteijn et al. (2001) in a driving simulation task. Presence measured by subjective responses (continuous scale) showed a large increase due to stereoscopic presentation. The authors also found that a behavioral measure of presence, postural sway, also showed a tendency to increase when stereoscopic cues were added. Importantly, the authors also measured sickness in this study and identified no effect of stereoscopy on VIMS (continuous scale), although it should be noted that sickness ratings were near floor levels. These results were similar to those obtained by Ling et al. (2013), who found no difference in CS (SSQ) across stereoscopic and non-stereoscopic display conditions.

### Vection

Vection is considered a strong correlate of presence. For an observer to experience the illusion of self-motion, their sensorimotor control system must be convinced that the visual motion veridically specifies their own body motion (Prothero and Hoffman, 1995; Chertoff and Schatz, 2014). However, if the implied body motion is not consistent with cues from other modalities (particularly vestibular signals), sensory conflicts emerge, producing nausea (Reason and Brand, 1975; Lin et al., 2002). Vection-inducing stimuli are often nauseogenic, but the relationship is complex. Some have suggested that experiencing vection might be a necessary prerequisite for experiencing VIMS (Hettinger et al., 1990; Hettinger and Riccio, 1992; Keshavarz et al., 2015). Motivated by this hypothesis, one study has characterized an optimal magnitude of visual motion that does not produce CS but still evokes vection (Tanaka and Takagi, 2004).

However, vection does not always lead to the emergence of sickness symptoms. There is strong evidence that VIMS can occur in the absence of vection (Ji et al., 2009). Other studies have presented evidence that suggests a negative relationship between vection and CS (Bonato et al., 2008; Palmisano et al., 2017). Palmisano et al. (2017) recently found that individuals who felt stronger vection (magnitude estimates) were likely to report fewer symptoms of CS (SSQ). This effect was dependent on the visual display conditions: The relationship was only obtained when visual stimuli were observed through a simulated aperture (field-of-view: 86° diagonal), and not when participants observed a “full field” stimulus (field-of-view: 110° diagonal). The authors reiterated that the link between vection and CS was relatively weak, and that a complex relationship is likely to exist. In several other experiments, there appeared to be no association between VIMS and vection (Webb and Griffin, 2003; Keshavarz and Hecht, 2011a; Riecke and Jordan, 2015). Evidently, there are highly complex relationships between vection and CS, as well as between vection and presence. This complexity has been discussed by others who suggest that vection poses an intervening factor between presence and CS (Stanney et al., 1998; Sadowski and Stanney, 2002; Hettinger et al., 2014). Concurrent measurements of each variable will be essential in future research studies attempting to model the presence-CS-vection relationship.

### Intuitiveness of Interaction

Presence has been termed the illusion of a non-mediated experience (Lombard and Ditton, 1997). This illusion is encompassed by the absence of attention to the apparatuses used to convey a simulation, such as the visual display device itself, the environmental boundaries, and the controls used to interact with the environment. For this reason, natural (ecological) control methods that do not distract from the simulation are likely to produce higher presence. Examples of this principle were provided by Welch et al. (1996), who indicated that the ability to interact with the environment increases presence, and that increasing the latency between action and feedback can negatively impact presence. Additionally, Schuemie et al. (2005) showed that more “natural” locomotion techniques (i.e., walking in place compared with mouse navigation) lead to a greater sense of presence (IPQ). Equally, the intuitiveness of the control scheme in VR has been linked to CS rates, with a greater degree of CS provoked by less ecological control schemes. For instance, Kolasinski (1995) discusses that freezing or resetting the simulated viewpoint of an observer tends to be highly nauseogenic. Borrego et al. (2016) report that navigating a virtual environment by walking leads to increased levels of presence (SUS and PQ) compared with using a hand-held controller to navigate. Additionally, Jaeger and Mourant (2001) documented that navigating by walking on a treadmill led to significantly lower CS severity (SSQ) than when a hand-held controller was used. These series of findings are perhaps unsurprising, given that exposure to novel sensorimotor conditions in the real world is known to provoke sickness (e.g., prism glasses that reverse the orientation of the visual field are initially nauseogenic for users, Oman, 1991). However, some research has indicated that more intuitive controls do not affect CS (e.g., Borrego et al., 2016), or can even lead to an increase in CS (e.g., the addition of head tracking in the study of Schuemie et al. (2005), led to an increase in SSQ scores), although this may be related to the small sensory mismatches introduced by imperfect tracking conditions in these cases. It appears likely that presence and comfort are both reduced when interacting with a virtual environment in a manner that is unfamiliar in terms of sensorimotor control. As such, results of experiments that manipulate the control scheme in VR may tend to suggest a negative relationship between CS and presence due to the inverse effect of sensorimotor familiarity on each factor.

### Navigation Control

The capability of action within a virtual environment has frequently been linked to the feeling of presence in VR (e.g., Sanchez-Vives and Slater, 2005; Slater, 2009), and in line with this idea there is evidence that controlling one’s own locomotion in a virtual landscape increases presence (Stanney et al., 2002; Clemente et al., 2014). There is also a long history of research documenting the nauseating effects of being moved passively in VR and driving simulators (Rolnick and Lubow, 1991; Stanney and Hash, 1998; Sharples et al., 2008; Dong et al., 2011). Predictive movement control allows a user to compare estimated and obtained sensory data in a feedforward control loop, which is thought to reduce the impact of decoupling efferent and afferent signals (Reason and Brand, 1975; Rolnick and Lubow, 1991).

As part of a study by Seay et al. (2002) (described above), the authors investigated the effect of being the driver or the passenger in a driving simulation. Enacting the role of the driver increases the sense of presence (PQ). At the same time, the magnitude of CS was higher for passengers compared to drivers (SSQ), as found in other research (Rolnick and Lubow, 1991; Stanney and Hash, 1998; Sharples et al., 2008; Dong et al., 2011).

Results of a recent study have indicated that navigation in a virtual landscape (i.e., locomotion using an Xbox 360 Gamepad) increases presence (SUS) compared to conditions where participants remained relatively stationary (head tracking and motion parallax, but no locomotion), but that sickness scores (SSQ) are unaffected by the same manipulation (Ibánez and Peinado, 2016). This suggests that presence increases when participants are permitted to freely explore an environment, even if the navigation method used is relatively unnatural (i.e., navigating with a gamepad). However, the manipulation used by the authors cannot discern whether other mediating variables might have played a role, such as optic flow or vection produced by locomotion.

### Context

Narrative is often used to provide context and framing to a VR application, and there is evidence that the inclusion of narrative impacts both presence and CS. The influence of a “preamble” on presence has recently been established (Smolentsev et al., 2017): When participants initiate a VR task in a digital environment similar to their own physical location, the sense of presence (single item scale) increased significantly compared to when the digital environment portrayed a different physical location. The authors stated that the benefit of a familiar environment on the sense of presence is achieved by establishing a physical continuity between the user’s experience in the real environment and the virtual landscape.

The effect of context on presence has been frequently studied with respect to the mediating effect of anxiety on presence. There is thought to be a complex, potentially bi-directional relationship between presence and anxiety, with both being associated with general sympathetic nervous system activity (Rothbaum and Hodges, 1999; Krijn et al., 2004). Gorini et al. (2011) show a heightened sense of presence (SUS) if an anxiety-inducing narrative context was provided while the participant searched for an object in VR (i.e., the participant was being “chased” by a “murderous” person as they searched). A significant increase in presence (single item scale) also occurs if participants with a phobia are told that the virtual environment may contain a phobic stimulus (Bouchard et al., 2008). On the other hand, the use of a different measurement scale (PQ) has resulted in the opposite trend: Anxiety-inducing narrative context produced a reduction in total presence (Bouchard et al., 2008). Although the authors attributed this divergence in results to one or two items in the PQ dominating their results, this provides further evidence for a low level of reliability between common measures of presence. The effect of anxiolytic narrative on CS (SSQ) was also measured by Bouchard et al. (2008), with the authors finding no relationship between the two variables. A similar pattern of results was obtained by Robillard et al. (2003), who found a relationship between presence and anxiety (both single item scales) but no link between either factor and CS (SSQ), although CS scores were overall very low. The absence of a link between CS and anxiety is somewhat surprising, given that trait-anxiety measures partially determine the likelihood of motion sickness in the general population (Paillard et al., 2013), and as such future studies will need to investigate this link further.

### Sex

There has been considerable discussion about the effects of participant sex on presence ratings. Some have theorized that the degree to which men and women can suspend disbelief may vary (Slater and Usoh, 1994; Felnhofer et al., 2012), along with personality factors such as extraversion and submissiveness (Lombard and Ditton, 1997). Others have proposed that any sex effects on presence are likely due to the correlated differences in gaming experience between the sexes (Gamito et al., 2008, 2010). However, the empirical research is divided with respect to which sex demonstrates higher levels of presence. In an anxiety-inducing VR environment (a school examination), Gamito et al. (2008) reported evidence of a higher level of presence for women than for men (PQ realism), although the authors attributed this effect to the higher experience with video games among male participants. On the other hand, other studies have found that men report higher levels of presence than women in VR (Slater et al., 1998; Nicovich et al., 2005) and in non-VR video games (Lachlan and Krcmar, 2011). Felnhofer et al. (2012) documented evidence of a sex effect on presence ratings (IPQ spatial presence), with men rating themselves higher than women. Other research has found no difference between men and women with respect to spatial presence (De Leo et al., 2014).

Research into CS has long discussed the possibility of sex differences with respect to rates of susceptibility, although findings have proven inconclusive. In the work of Knight and Arns (2006); Gamito et al. (2008), and Ling et al. (2013) described elsewhere here, the authors failed to identify any difference in CS across the sexes. Conversely, studies by De Leo et al. (2014) and Jaeger and Mourant (2001) revealed that female participants were significantly more likely to experience CS symptoms than male participants. In a similar vein, Park et al. (2006) reported that non-dropout female participants exhibited more CS symptoms (SSQ nausea and oculomotor subscales) than male participants did in a non-immersive driving simulator. Häkkinen et al. (2002) also found CS ratings (SSQ) were lower for men than for women. Some have also suggested that the reason for the discordant findings on sex and CS may relate to hormonal changes across the menstrual cycle, resulting in a fluctuating relationship (Biocca, 1992; Clemes and Howarth, 2005).

### Gaming Experience

Some research has examined the influence of past experience with interactive games on factors such as presence and CS. Knight and Arns (2006) identified an inverse association between an individual’s experience playing video games and the level of CS experienced. Various studies report no relationship between presence and gameplay experience (Schuemie et al., 2005; Alsina-Jurnet and Gutiérrez-Maldonado, 2010; Ling et al., 2013). Others have found minimal evidence for an effect of video game experience on presence ratings or CS. Gamito et al. (2010) experimentally manipulated gameplay experience using a training procedure, and reported that increased gaming experience leads to improved presence (PQ) whereas CS (SSQ) was unaffected by training. In another study that employed a similar task, Gamito et al. (2008) found no relationship between measures of presence (PQ, ITQ) or CS (SSQ) and the previous gameplay experience of participants. At the same time, the authors found an increase in physiological markers of heart rate with increasing experience with video games. Those authors considered heart rate as a measure of anxiety, but we note that others have taken similar markers to indicate presence (e.g., Meehan et al., 2001, 2003; Wiederhold et al., 2001) and also CS (e.g., Kim et al., 2005; Dennison et al., 2016). Accordingly, one should be cautious when interpreting physiological markers purported to measure these factors given the relative paucity and inconsistency of data of this sort reported to date.

### Conclusion: Associated Variables

When taken together, the evidence obtained from the review above begins to clarify the type of relationship that exists between presence and CS:

• Approaches that reduce sensory mismatch show potential for reducing CS and increasing presence;
• Both presence and CS are increased by the addition of stereoscopy, high field-of-view display conditions, and by enhancing the likelihood that a display will evoke vection;
• Increasing factors such as intuitiveness of interaction and control of navigation lead to higher presence and lower CS;
• Men and individuals with more gaming experience demonstrate lower CS and higher presence, although the partial effects of sex and gaming are not fully clear.

The relationship between CS and presence can be understood if the associated variables described above are considered with respect to their effect on sensory mismatch. Immersiveness (sensory submersion) likely plays a key role here: Experimental manipulations that increase immersiveness tend to produce both CS and presence, because the compelling nature of stimuli in an immersive virtual space fosters a high perceptual weighting of cues to self-motion and spatial orientation, which enhances the impact of conflicts between expected and obtained sensory signals that are generated by the compelling stimulus (Prothero, 1998; Lombard et al., 2000; Prothero and Parker, 2003). Put differently, immersiveness enhances the magnitude of violated expectations. Thus, increasing field-of-view size, adding stereoscopy, or providing congruent multisensory information can increase both presence and CS. Given that immersiveness (which increases the weight of sensory conflicts, Prothero, 1998) can also lead to increased vection (which is inversely related to sensory conflicts, e.g., Weech and Troje, 2017) it is unsurprising that research on the vection-presence-CS relationship has concluded that the link is extremely complex (Keshavarz et al., 2015; Palmisano et al., 2017).

On the other hand, within immersive conditions, individuals who experience high presence tend to experience low levels of CS. This relationship may be a result of differences in individual sensitivity to sensory conflicts: Higher sensitivity will result in high CS and low presence, whereas low sensitivity to cue conflicts will lead to low levels of CS and a heightened sense of presence. Individual differences in sensitivity to sensory conflicts have been documented, and there is some limited evidence that these differences relate to CS and presence (Viaud-Delmon et al., 2000, 2006). The advantage in terms of presence and CS observed for “gamers” may be attributed to the process of sensory reweighting that occurs during continuous exposure to cue conflicts (see Reason and Brand, 1975; Weech et al., 2018a). Indeed, a sex effect on presence and CS possibly relates to the superior ability of men to adapt to sensory conflicts compared with women (Viaud-Delmon et al., 1998). Further research on these individual differences will be required in order to test the proposition that variance in sensory conflict sensitivity underlies the experience of both presence and CS.

There is a significant challenge involved in identifying the factors that might mediate the link between presence and CS. Primarily this difficulty arises from the substantial relationships between the associated factors identified above, such as sex and gameplay experience, or vection and field-of-view. In addition to these known relationships, a number of other understudied variables may have significant associations with both presence and CS. For instance, the sense of embodiment is known to form a core aspect of presence (Kilteni et al., 2012), but the relationship between the embodiment of a virtual body and CS is currently unclear. Furthermore, questions remain about how prior experience with VR systems interacts with presence and CS; experiential factors are currently understudied due to the novelty of widely available VR technology. It is also evident that there is a lack of research that combines measurements of presence, CS, and other factors in high-powered studies. The collection of large datasets that encompass multiple individual factors (age, sex, personality type) with several behavioral response measures (objective and subjective measures of presence and CS) permits the use of a modeling approach that would enhance our understanding of the complex relationship between presence, CS, and other mediating factors (a similar approach was adopted for CS alone in Weech et al., 2018b). Through the execution of such studies in the future, further interactions will be uncovered between the associated factors outlined above.

Questions remain with regard to what questionnaires of presence and CS are truly measuring. Studies have identified relationships between CS and spatial presence, but no relationship between CS and immersive tendencies, which correlates highly with spatial presence (e.g., Ling et al., 2013). This raises the problem that the degree to which an individual’s tendency to *report* feeling presence or CS may not determine their *experience* of either factor. This is an inherent issue in questionnaire studies; according to a meta-analysis, almost 50% of questionnaire studies documented effects of social desirability on their results (Van de Mortel, 2008). It is therefore important that future research takes into account the possibility that response bias modulates measures of factors like presence and CS. Several approaches could be adopted to achieve this, including pre-task questionnaires that assess social desirability (Crowne and Marlowe, 1960; Van de Mortel, 2008) or developing and using questionnaires according to principles of minimizing bias (Choi and Pak, 2005).

## General Conclusion and Future Directions

Literature supports the idea that presence and CS are inversely related, and that the relationship is likely to be mediated by factors including vection, navigation control, and display factors. These factors can be unified in terms of their effect on sensory mismatch, which appears to drive presence and CS in opposite directions. This presents the possibility that interventions targeted at increasing presence could reduce CS, and vice versa. While the results obtained across studies are often discordant, with many sources reporting a positive relationship between presence and CS, these outcomes may be related to the fact that immersive displays are likely to generate both a compelling sense of “being there,” as well as symptoms of physiological discomfort. Other noise sources that may contribute to variability in findings include problematic measurement techniques, or differences in the operational definition of the underlying factors among studies.

How can future experimentation best serve the advancement of our understanding of the presence-CS association? The issue of measurement validity must be a major focus of future studies, where the cross-validation of metrics should be undertaken in well-controlled paradigms. Improving the robustness of findings in this area may also require a careful consideration of factors that could play a role in response bias, such as social desirability. Although a limited number of high quality studies have collected large datasets related to human factors in VR, future experiments will need to combine these measures with a modeling approach that can help to interpret the structure of the relationship between these factors. Relatedly, there is a prevalent lack of statistical power in many of the studies reviewed here, and this limits the ability for the field to infer answers about variables that are so inherently noisy. Future studies will benefit from careful *a priori* considerations of effect sizes, which we have compiled here where available. An additional factor that may reduce the variability in findings across future studies is the natural emergence of standardized VR head-mounted hardware. Note, however, that the findings of studies using lower-immersive systems such as projection screens will still prove valuable, as these will help to identify the impact of immersiveness and vergence-accommodation mismatch on CS and presence. One particular gap in our understanding revealed by the current review is how presence is affected when sensory mismatch is experimentally manipulated. Given the prospective modulatory role of sensory mismatch in the association between presence and CS, future studies will need to overcome the challenges in manipulating and assessing sensory mismatch in empirical research. Through a careful consideration of the literature critique provided here, we envisage that the next wave of studies on the presence-CS link will help to make major advances toward understanding this complex relationship.

## Author Contributions

SW, SK, and MB-C designed the concept of the article and conducted revisions to the manuscript. SW and SK conducted the literature search. SW wrote the initial draft of the article.

## Conflict of Interest Statement

The authors declare that the research was conducted in the absence of any commercial or financial relationships that could be construed as a potential conflict of interest.

## Footnotes

1 Although non-immersive desktop systems are sometimes referred to as “virtual reality,” we use the term here to signify immersive or semi-immersive systems such as head-mounted displays or projection systems.

2 Note that this review includes discussion of several related phenomena, such as CS, visually-induced motion sickness (VIMS), and simulator sickness (SS). Due to the important differences between these phenomena (which we briefly discuss below), the reader is advised to consider that evidence of a relationship between vection and VIMS, for example, does not dictate the same association between vection and CS.
